# Integrated Optical Deformation Measurement with TIR Prism Rods

**DOI:** 10.3390/s23020943

**Published:** 2023-01-13

**Authors:** Alexander Wolf

**Affiliations:** 1 Institute of Product Development, Gottfried Wilhelm Leibniz Universität Hannover, An der Universität 1, 30823 Garbsen, Germany; wolf@ipeg.uni-hannover.de; 2 Cluster of Excellence PhoenixD (Photonics, Optics, and Engineering—Innovation across Disciplines), Welfengarten 1A, 30167 Hannover, Germany

**Keywords:** optical measurement, integrated measurement, prism rods, deformation, torsion, condition monitoring, total internal reflection, decency level of sensitivity

## Abstract

In this paper, a novel optical measurement principle for deformation, especially torsion, is presented. A laser beam is guided via total internal reflection (TIR) in a prism rod. Every single reflection causes an increasing change in the beam path, which can be measured by its effect on the outcoupling position of the laser. With a diameter of the prism rod of 10 mm and a length of 120 mm, the system achieves torsion sensitivities between 350 µm/° and more than 7000 µm/°, depending on the actual torsion angle φ. A decency level of sensitivity is defined for comparison, which is exceeded by a factor of ~55 at φ=0. The presented principle of TIR prism rods can be adapted to measure different load cases. Using two laser beams, bending and torsion can be distinguished and combined load cases analyzed. The resulting system can be integrated into machine elements, such as screws, to perform condition monitoring on mechanically loaded components.

## 1. Introduction

For structural components that are used under variable environmental conditions or have to withstand uncertain loads, it is useful to monitor the installed joints. This can be done by regular inspection or by using measurement technology integrated into the system. In this paper, an approach to equip a standard machine element, such as a screw, with sensor functionality is presented. In this way, the same machine element can be used for different applications without the need for adaptation. In addition, one device to be monitored can be equipped with several “sensor” screws to detect complex load situations.

Screw joints are usually based on pretension of the connected elements. In this process, the screw is elongated and twisted. This load situation changes if the preload decreases over time due to external influences, due to a failure of the screw itself or of neighboring components. The data obtained through this monitoring allows for the understanding of complex load situations of mechanical structures [1] and can be used to optimize subsequent product generations [2,3]. Conventional approaches for measuring the axial force in a screw joint are based on additional sensor elements, such as washers with deformable structures, which are standardized in EN 14399-9 [4]. Since the deformation of these washers is measured by hand, they only allow limited resolution and lack the ability for automated condition monitoring. For a screw equipped with a washer-like disk which changes its electrical resistance under mechanical stress, a high sensitivity is reported [5].

Another approach is to directly integrate the sensor into the screw. This can be done by using strain gauges [1] or by integrating an electrical resonant circuit whose resonant frequency changes with the deformation. Here, a sensitivity for deformation in the range of a few tenths of a millimeter is reported for an M24 screw [6]. Both examples are based on additive manufacturing of the screw, which—due to the lack of standardization of these processes and the materials used—leads to a poorly definable mechanical load capacity of the screw. Another possibility is to abandon the mechanical function of the screw and only use it as housing for a sensor [7]. Further approaches to integrate a sensor in a machine element are currently under research as part of the SPP 2305, funded by the German Research Foundation (Deutsche Forschungsgemeinschaft DFG) [8,9].

In this paper, a measurement principle based on total internal reflection of a laser beam is proposed (Section 2). In contrast to other optical measurement approaches like interferometry or structured light scans, this does not require a complex setup. Optical measurements are easily influenced by environmental conditions, such as dust or external radiation sources [10]. Many of these factors can be avoided by integrating the sensor system into the measurement object, which thus encloses the light emission, beam path and detection. Due to this encapsulation, a robust system can be realized. Using the example of a screw, measuring torsion is of particular interest to determine the pretension of the connected parts (see discussion in Section 2). For other machine elements, bending is also relevant. Therefore, torsion measurement is considered in Section 3.1 while bending is handled in Section 3.2. The superposition of both load cases is the focus of Section 3.3. The energy supply of the sensor and the data transmission between the sensor and the environment are not the focus of this paper. Approaches to solving this can be found in the literature [6,7,11].

## 2. Measurement Principle and Simulation Setup

To monitor the load situation in the screw, a laser-based measurement system is developed that provides high sensitivity to torsion without the need for precise adjustment. The laser beam should not interact with the surfaces of the screw itself. In this way, the requirements for manufacturing the cavity inside the screw are not too high. Instead, a polymer multimode fiber is integrated into the screw that guides the laser and can be easily manufactured with high surface quality. Due to the mechanical connection between fiber and screw, both components are deformed together. Using an appropriate setup, this can be detected as modulation of the light intensity, polarization, wavelength, refractive index, propagation time, coherence length or of the light path itself [12].

A well-known approach to measuring the deformation of such a fiber is based on Bragg gratings [13,14]. They are very sensitive to elongation, which results in a change in the grating period. Therefore, Fiber-Bragg gratings (FBGs) can be used to detect strain and temperature changes with high sensitivity. To differentiate between both parameters, two different FBGs, chirped FBGs [15] or a combination of an FBG with a long-period grating can be used [16]. Further approaches can be found in the literature [17]. A three-dimensional array of FBGs can serve as a high-sensitive sensor system for 3D shape measurements [18,19], e.g., by using multiplexing technologies [20]. The disadvantage of Fiber Bragg gratings is that they require a precise evaluation of the outcoupled or reflected wavelength(s) to measure the elongation. This is typically realized with a diffractive optical element like a grating in combination with a linear CCD or CMOS sensor. Since these components have to be arranged at certain distances to each other, it is a challenge to fit the entire measurement system into a part of limited size, like a screw.

Another strategy is based on total internal reflection. Using a source that emits radiation under different angles, rays are coupled into a multimode fiber. Deformation, especially bending, increases the number of rays coupled out of the fiber and, therefore, decreases the signal intensity at its remote end. By applying the optical fiber in a curved shape onto the measurement object, length changes can also be measured [21]. While this measurement principle is quite cost-efficient, it requires exact knowledge of the intensity of the coupled radiation. Therefore, it is not very sensitive to small geometry variations and is easily influenced by aging processes and temperature changes.

A third established fiber-based measurement principle is interferometry (see review paper from Li et al. [22]). Due to the high requirements, e.g., on the stability of the emitted wavelength, it is not considered further here.

A fairly inexpensive approach is to map a structured light pattern onto a CCD or CMOS sensor. The pattern is shifted or rotated on the detector depending on the deformation of the surrounding structure, which can be quantified with image correlation. Al-Baradoni and Groche developed a measurement system based on this approach, which can be integrated into a cylindric machine element to detect forces in different directions, as well as torsion [23,24]. The disadvantage is the limited sensitivity of the system.

To find an effective measurement principle, multi-pass cells are considered. Here, a laser beam is reflected several times between two concave mirrors to quantify the absorption of the fluidic medium in between [25,26,27]. Due to the number of reflections, tolerances in the alignment of the mirrors are critical [28], as they typically add up for each reflection and lead to significant changes in the positions of the laser beam on the mirrors. This effect is undesirable since, after a defined number of reflections, the laser has to pass through a small hole in one of the mirrors to reach the detector. However, in the case considered here, such a system where small changes in position or orientation significantly modify the beam path is advantageous. The idea of amplifying small angular changes via multiple reflections and thus making them measurable is not new. Huang et al. [29] presented an amplitude-based two-dimensional angle measurement setup. This was improved by Huang and Ni [30] using multiple reflections. Chiu et al. [31] also used multiple reflections to improve the phase measurement setup from Chiu and Su [32].

**Figure 1:**
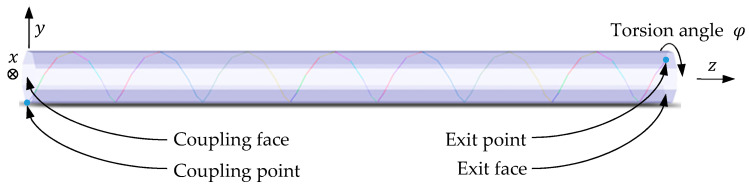
Spiral-shape total internal reflection of an ideal laser beam in a prism rod with eight lateral faces. Each reflection is indicated with a change of the beam color.

A screw under load is deformed by torsion (twist deformation) and pulling force (elongation and cross-sectional reduction). To measure the screws pretension, it is sufficient to detect one of these deformations. While elongation does not change the orientation of the faces of the fiber integrated in the screw, torsion does. Therefore, the idea of using multiple reflections to achieve high sensitivity to angular changes is applied to torsional deformations. This requires a three-dimensional setup. A multimode fiber with a regular polygon as cross-section—a so-called prism rod—is used to guide a well-collimated laser beam. The laser is coupled into the rod in such a way that it is reflected several times on the lateral faces based on total internal reflection, and thus, propagates in a spiral shape (Figure 1). A torsion of the rod around the z-axis affects the angular orientation of each lateral face. Therefore, the incidence angle of the laser beam at an exemplary lateral face is changed and the position at which it hits the next face is also modified.

**Figure 2:**
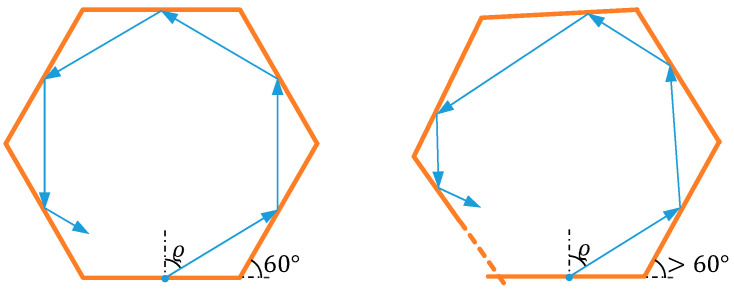
Reflections in a polygon. **Left**: regular polygon, **right**: polygon with deviations of the corner angles. The effect on the beam propagation is strongly exaggerated.

An efficient and sensitive measurement system can be designed when the deviations in angle and position increase with each reflection and significantly influence the outcoupling position of the laser beam. To verify this assumption, the situation is simplified to a two-dimensional problem (Figure 2). The reflection of a ray in the polygonal cross-section of the rod is considered, ignoring its propagation along the z-axis. While the ray starts in the middle of one edge and also hits the neighboring edge in its middle in the undeformed case, a specific change is applied to all corner angles (Figure 2, right image).

**Figure 3:**
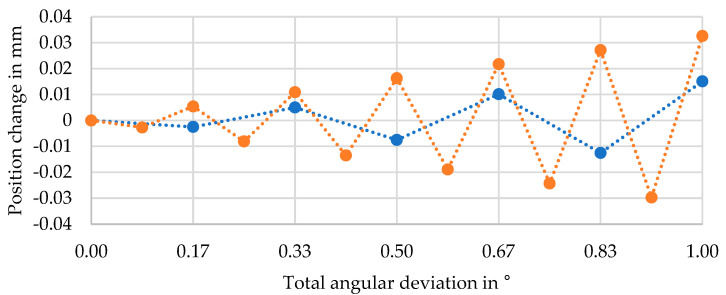
Position changes for reflections in a polygon with deviations in the corner angles. Blue: hexagon, orange: dodecagon.

For the polygon being a hexagon, the angular deviation is 1/6° per corner, which sums up to 1° for one full circle of the laser beam (Figure 3, blue dots). Similarly, the angle change per corner is 1/12° for a dodecagon, which also leads to 1° in total (Figure 3, orange dots). The distance between the two opposite corners is d=10 mm for the undeformed polygon. Figure 3 shows that the position where the laser hits the edges changes with alternating signs and an almost linearly increasing intensity. The resulting change in position for a full circle is greater for twelve edges than for six edges, and is, therefore, generally assumed to be greater as the number of edges increases.

**Figure 4:**
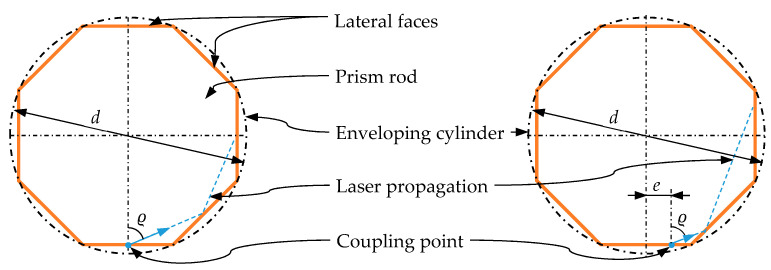
**Left**: coupling point of the laser at the first face of the prism rod (“central coupling”). **Right**: decentral coupling (this will be discussed in Section 3.1.3).

These 2D results cannot be fully transferred to the three-dimensional rod, since its plane lateral faces are not only tilted under torsion, but also curved. However, the results show that changes in position due to deformation add up for each reflection. Similarly, a large number of reflections is expected to be advantageous in the three-dimensional case to increase the measurement sensitivity for torsion. In a first step, the laser is coupled into the rod at the center of one lateral face (Figure 4, left image). All of these cross-section views, such as in Figure 4 or, later on, the detector views, are seen in the negative z-direction. Therefore, the horizontal axis is the positive x-direction and the vertical one is the positive y-direction (compare coordinate system in Figure 1). According to Equation (1), with n being the number of faces, the laser propagates to the center of a neighboring face.

(1) $$\varrho =90{}^{\circ}-\frac{180{}^{\circ}}{n}$$

Depending on the coupling angle ε, the laser beam travels a certain distance in z-direction on route. The angle ε is measured between the laser beam and the normal of the coupling face, ε=0 means propagation parallel to the z-axis. The length of the rod is 120 mm. For the numerical stability of the simulation, the laser is coupled in the rod at z=0.0001 mm, while the detector is placed at z=199.9999 mm, which reduces the effective rod length to l=119.9998 mm. The thickness of the rod is defined via the diameter of the enveloping cylinder with d=10 mm (compare Figure 4).

The CAD geometry of the rod is set up with Autodesk Inventor, while Zemax OpticStudio is used for ray propagation. Preliminary tests have shown that using an exchange format like *.stl leads to significantly wrong results, even with high resolution of the tessellation. Therefore, the Autodesk Inventor link from Zemax OpticStudio is used for data exchange. For the same reason, an FEM calculation of the deformed geometry is omitted. Instead, the following assumptions are made:

- Torsion and bending do not alter the cross-section of the rod.
- Torsion does not change the length of the rod.
- For bending, the arc length of the neutral fiber (the middle of the rod) is constant.

For torsion, these simplifications are of greater importance when the number of lateral faces n is small. As the number of faces increases, the cross-section becomes more and more similar to a circle, and the influence of these simplifications diminishes.

The material of the rod is defined as PMMA and the laser wavelength as λ=550 nm. This leads to a refractive index of the rod of $n_{\mathrm{rod}}=1.494$, which is assumed to be constant. The surrounding material is assumed to have the refractive index $n_{0}=1$. These optical parameters are not of great interest as long as:

- total internal reflection under the considered angles is possible;
- material absorption and scattering for the laser wavelength are low.

Therefore, a cladding with $n_{0}<n_{\mathrm{cladding}}<n_{\mathrm{rod}}$ can be used to mechanically connect the rod and the screw. For the considered wavelength (λ=550 nm), an optical loss of approximately 100 dB/km for PMMA fibers is reported [33]. For the sensor system, this attenuation is not critical. In Section 3, coupling angles between 60° and 80° are considered. For an angle of ε=60°, the propagation distance of the laser in the rod is 240 mm. This leads to an energy loss of 0.6% due to absorption. An angle of ε=80° results in a distance of 691 mm and an energy loss of 1.6%.

The diameter of the laser beam is assumed to be infinitesimally small. Therefore, the whole approach can be scaled. The simulation using Zemax OpticStudio is based on ray tracing, the validity of which must be verified. In general, ray tracing is suitable when the fiber diameter is significantly larger than the wavelength under consideration. With d/λ≈18,000, this condition is fulfilled here. The number of modes M that can propagate in the rod can be used as additional evaluation criterion.

(2) $$M\approx \frac{1}{2}V^{2}=\frac{1}{2}{\left(\frac{\pi d^{*}}{\lambda }\sqrt{n_{1}^{2}-n_{0}^{2}}\right)}^{2}$$

In Equation (2), V is the normalized frequency of the rod. Since this equation is designed for circular fiber cross-sections and the rod geometry does not fill the whole circle with diameter *d* (compare Figure 4), the reduced value $d^{*}=9.5\;\mathrm{mm}$ is used here as an approximation. This leads to a mode number of $M\approx 3.4\cdot 10^{8}$. For this high number, it is assumed that individual modes do not have to be considered and that ray tracing is suitable to describe the light propagation in the rod.

Birefringence is an important factor for optical polymers, especially when it comes to deformation. For the proposed sensor system, this effect can either be minimized or used as part of the detection principle. There are already approaches to reduce or even compensate the birefringence of PMMA, taking into account both static orientation birefringence and deformation-induced photoelastic birefringence [34] (also compare [35]). In this way, a sensor system with minimal birefringence can be realized. Accordingly, the effect of birefringence is ignored in this paper. A specific use of birefringence as part of the measurement approach is presented in Section 4.

The larger the coupling angle ε, the more reflections occur in the prism rod. In order to couple the light into and out of the rod at large angles of ε, special treatment of the prism end faces is required. By either adding a small triangular wedge to the coupling face or removing a similarly shaped part from the volume, the angle at which the beam hits the polymer material can be reduced so that it is orthogonally incident if desired. Due to the measurement principle, the position at which the beam exits the rod is not constant. In order to be able to detect the laser beam at any point on the exit face, this face should be completely covered with the detector. The entire exit face should be roughened or have a prismatic structure, where the prisms are smaller or equal in size to the detector pixels. In this way, the laser can be coupled out of the rod at any point on the exit face and reach the detector. Since these aspects are relevant for the realization of a sensor but not to investigate measurement principles, they are not further considered in this paper.

**Figure 5:**
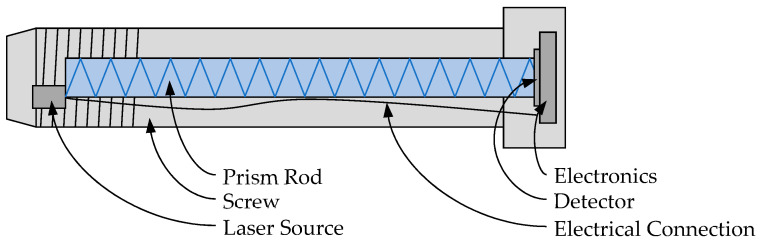
Concept of the sensor system integrated in a screw.

Figure 5 illustrates a possible realization of the sensor concept. The mechanical load of a screw under torsion and bending is greatest near the thread. If the sensor is placed along the longitudinal axis of the screw, it weakens its mechanical properties the least. A similar approach, in which additional functions are integrated into a mechanically loaded component at low-load points, is used in particle damping [36,37]. Since the modulus of elasticity of a screw, which is a metallic material, is significantly greater than that of the polymer rod, the deformation can be transferred from the screw to the rod without significant resistance. Depending on the screw type, the screw head offers more installation space than the opposite side. Therefore, the detector and electronics can be positioned in the screw head, while the laser source is located at the other end of the rod. The detector is positioned at the exit face and is assumed to follow its movements. Preferably, the rod extends over as much of the screw’s length as possible to maximize system sensitivity. All components are assumed to be perfectly aligned. The influence of misalignment is discussed in Section 3.4, and the effect of temperature changes is discussed in Section 3.5.

## 3. Results

### 3.1. Torsion Measurement

As described above, torsion of the prism rod changes the incidence angle of the laser beam on each lateral face. In this way the position of the laser beam at the end face changes as a function of the torsion angle. As not indicated otherwise, the considered incidence angle is ε=80° and the number of faces n=8. The prism rod has a diameter of d=10 mm and an effective length of l=119.9998 mm. According to Equation (3) this leads to a sum of 192 total internal reflections in the undeformed rod.

(3) $$n_{r}=\frac{2\cdot l}{d\cdot \sin\frac{2\pi }{n}\cdot \tan\left(\frac{\pi }{2}-\varepsilon \right)}$$

Please note that the brackets x stand for the floor function.

#### 3.1.1. Central Coupling

**Figure 6:**
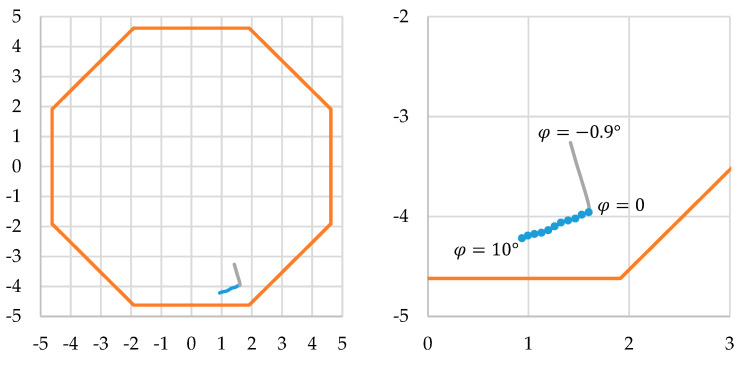
Position of the laser beam at the exit face in dependence of the torsion angle. Blue: 10°≥φ≥0, grey: 0≥φ≥−0.9°, orange: shape of the exit face. Dots in enlarged diagram for each full degree of φ. Dimensions are in mm.

The laser beam is coupled into the rod at the middle of a lateral face (see Figure 4, left). The position where it leaves the rod at the opposite face strongly depends on this coupling position and on the whole geometry. In this example (n=8, ε=80°, d=10 mm, l=119.9998 mm) the exit point is quite far away from any lateral face for the undeformed rod (φ=0) (Figure 6). As the torsion angle increases (φ>0), the exit point moves on a slightly wavy line (blue curve). A torsion of φ=0.1° results in a deviation of the exit point position of p=8.1 µm. This relatively small distance corresponds to 1.61 pixels assuming an exemplary pixel size of the detector of 5 µm × 5 µm. Here, for simplification, a laser movement in the direction of a pixel edge is assumed.

Twisting the rod in the other direction (φ<0) results in a different, almost straight path of the exit point (grey curve). For φ<−0.9°, the laser beam is coupled out of the rod before it reaches the exit face and, therefore, gives no signal on the detector.

The spacing of the dots in Figure 6 (enlarged image on the right), which mark full degrees of φ, is not constant. While they are in close neighborhood for φ>0 (blue curve), the grey curve (opposite direction) only goes to φ=−0.9° and, therefore, does not show a single dot. This indicates that the sensitivity of the sensor system strongly depends on the actual torsion angle φ. The local sensitivity σ of the system is determined according to Equation (4) by comparing the position $P_{i}$ of the exit point of the laser for one torsion angle with the neighboring points $P_{i+1}$ and $P_{i-1}$ (one in a positive and one in a negative direction) and dividing it by the difference of the torsion angles.

(4) $$\sigma_{i}=\frac{1}{2}\left(\frac{\left|P_{i+1}-P_{i}\right|}{\varphi_{i+1}-\varphi_{i}}+\frac{\left|P_{i}-P_{i-1}\right|}{\varphi_{i}-\varphi_{i-1}}\right)$$

**Figure 7:**
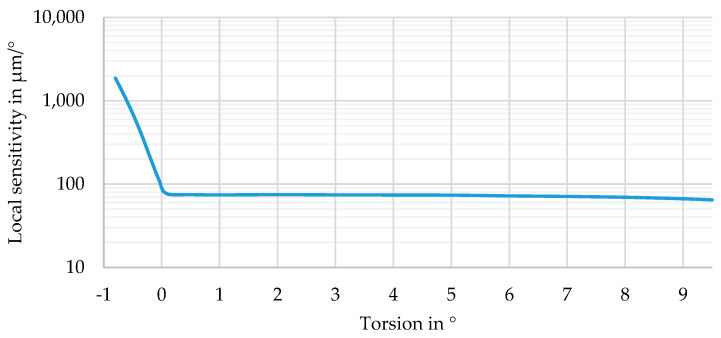
Local sensitivity for torsion.

A high sensitivity is achieved for negative angles of *φ* (Figure 7). The sensitivity almost exponentially decreases as φ rises from −0.9° to 0. At φ=0.1°, the sensitivity is σ=77 µm/° and slightly drops from there on. For φ=9.5°, the sensitivity is σ=65 µm/°.

The influence of the incidence angle ε and the number of faces n on the local sensitivity at φ=0 is summarized in Table 1. While increasing the incidence angle ε from 60° to 80° slightly raises the sensitivity for n=8, its influence at n=6 is not ambiguous. Except for ε=70°, the greater number of faces leads to an increased sensitivity.

#### 3.1.2. Decency Level of Sensitivity

To evaluate the efficiency of the measurement approach, a so-called decency level of sensitivity $\hat{\sigma }$ is defined. This idea of objectifying the results goes back to an approach that makes the image quality of camera lenses comparable, regardless of the camera body (and thus, the sensor) used [38].

The simplest possible measurement system is defined as the reference, which uses the same volume as the real one, but with a simplified measurement principle. The available space is a cylindrical volume with d=10 mm and l=119.9998 mm. The reference system, inspired by the sensor presented by Al-Baradoni and Groche [23], is a laser beam that starts at z=0, propagates parallel to the z-axis and hits a detector at z=119.9998 mm so that it does not undergo any reflection. When the planes z=0 and z=119.9998 mm are rotated against each other, the position of the laser beam on the detector changes. This effect is proportional to the distance between the laser and z-axis. For a fair comparison, the maximum distance d/2=5 mm is used to determine $\hat{\sigma }$. To keep the expression easy to understand, the term radian per degree is added. Therefore, the decency level of sensitivity $\hat{\sigma }$ is independent from the actual torsion angle φ.

(5) $$\hat{\sigma }=\frac{d}{2}\cdot \frac{\pi }{180{}^{\circ}}=87\;µm/{}^{\circ}$$

This value is in the range reached by the investigated system for φ≥0 (Figure 7). The factor $\sigma /\hat{\sigma }$ (see Table 1) can be considered as the gain of the measurement principle and should be significantly larger than 1. Therefore, an approach to increase the torsion sensitivity is presented in the following subsection.

#### 3.1.3. Coupling with Lateral Offset

**Figure 8:**
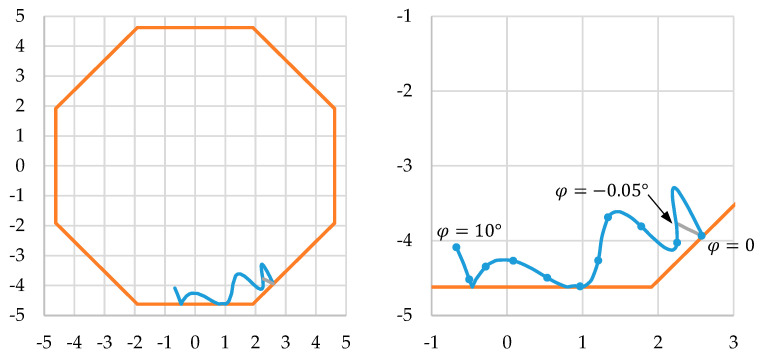
Position of the laser beam at the exit face in dependence of the torsion angle. Blue: 10°≥φ≥0, grey: 0≥φ≥−0.05°, orange: shape of the exit face. Dots in enlarged diagram for each full degree of φ. Dimensions are in mm.

**Figure 9:**
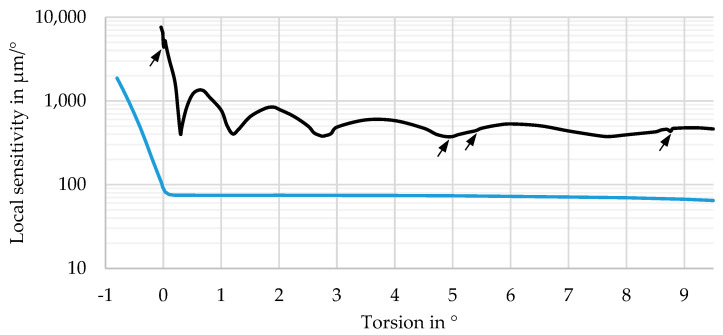
Local sensitivity for torsion. Black: lateral offset e=1 mm, blue: e=0 mm.

The sensitivity of the sensor can be strongly increased by moving the coupling point of the laser away from the middle of a lateral face (Figure 4, right). The angles ε and ϱ are the same as in Section 3.1.1. With a lateral offset e of 1 mm, the results are given in Figure 8 and Figure 9, as well as in Table 2. Additionally, the exit point of the laser strongly depends on the whole geometry. For the chosen parameters and without torsion (φ=0), it is located close to a lateral face and, therefore, at an edge of the rod (Figure 8). With increasing torsion (blue curve), the exit point moves on a curved path and again hits an edge of the rod at torsion angles of φ≈5°, 5.5° and 8.75°. Between φ=5° and 5.5° and above 8.75°, the number of reflections is reduced by 1 to $n_{r}=191$. A torsion of φ=0.1° results in a deviation of the exit point position of p=439.3 µm (equates to 87.86 pixels). Twisting the rod in the other direction (φ<0) results in a different path of the exit point (grey curve in Figure 8) and an increased number of reflections ($n_{r}=193$). For φ<−0.05°, the laser beam is coupled out of the rod before it reaches the exit face.

Compared to the system with central coupling (e=0), the lateral offset strongly increases the sensitivity (Figure 9). However, this measure reduces the useful measurement range from φ>−0.9° to φ>−0.05°. The curve for e=1 mm (black) roughly shows an exponential decay for −0.05°<φ<0.3° and, from there, a wavelike behavior. The maximum sensitivity is σ>7000 µm/°. For all considered angles, the local sensitivity is σ>350 µm/° and, therefore, is at least four times the decency level $\hat{\sigma }$ (see Section 3.1.2).

The instability at φ=0 corresponds to the exit point of the laser hitting an edge of the rod. This also happens for the above-mentioned angles of φ, and is indicated with arrows in Figure 9. The local minima of the black sensitivity curve correspond to the points where the path of the exit point in Figure 8 shows a strong curvature.

The local sensitivity at φ=0 for different numbers of faces n and coupling angles ε is given in Table 2. Other than for central coupling, the sensitivity clearly corresponds to the number of reflections $n_{r}$ and can, therefore, be increased by

- increasing the number of faces n,
- increasing the coupling angle ε, both shown in Table 2, or by
- increasing the rod length l while keeping the torsion per length φ/l constant.

The sensitivity for decentral coupling is significantly higher than for central coupling and reaches up to 55 times the decency level $\hat{\sigma }$ for φ=0. Therefore, the system can be considered to have a high sensitivity to torsion.

### 3.2. Bending Measurement

In just a limited number of applications, torsion is the only load case. It is often combined with a bending moment. In this section, the influence of bending is considered for the most sensitive geometry analyzed so far (same parameters as in Section 3.1.3; n=8, ε=80°, e=1 mm). The position of the coupling point remains unchanged at the edge of the rod. The bending direction is defined with the angle α, while α=0 stands for bending around the y-axis (the remote end of the rod moves in positive x-direction). The angle β defines the intensity of the bending and is the angle between the coupling face and the exit face.

**Figure 10:**
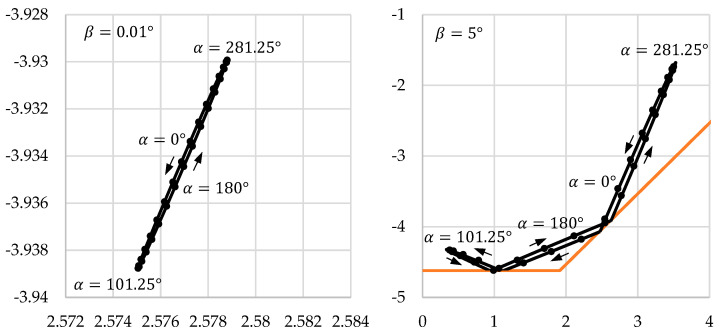
Position of the laser beam at the exit face in dependence of the bending orientation α. One dot each 11.25° of α. Left: bending angle β=0.01°, right: β=5°. Dimensions are in mm.

**Figure 11:**
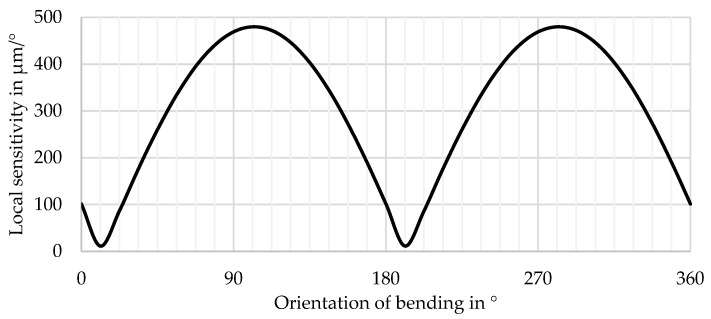
Local sensitivity of the sensor system $\sigma_{B}$ for small bending angles β in dependence of the bending orientation α.

For small bending angles β, the exit point moves on an elliptical path when the bending direction α is rotated (Figure 10 left). For larger bending angles, the exit point may hit an edge of the rod so that the “bending ellipse” is folded (in Figure 10, right image, it is folded twice). Due to the narrow shape of the ellipse the sensitivity is small for two specific rotation angles (here, α≈12° and α≈192°) while it reaches maxima of $\sigma_{B}\approx 480\;µm/{}^{\circ}$ for the two angles in between (α≈102° and α≈282°) (Figure 11).

To determine the decency level of sensitivity $\hat{\sigma_{B}}$ for bending, the same approach as in Section 3.1.2 is used. However, in order to obtain results independent from the orientation of the bending direction (angle α), the reference laser beam propagates along the z-axis (starting point x=y=0). The decency bending sensitivity

(6) $$\hat{\sigma_{B}}=\frac{l}{\beta^{2}}\cdot \left(1-\cos\beta \right)\cdot \frac{\pi }{180{}^{\circ}}$$

can be approximated with the constant value

(7) $$\hat{\sigma_{B}}\approx \frac{l}{2}\cdot \frac{\pi }{180{}^{\circ}}=1047\;µm/{}^{\circ}$$

for small bending angles β. All sensitivities presented in Figure 11 are significantly below this value. Nevertheless, bending can be measured with the system and will influence the results for torsion in combined load cases.

**Figure 12:**
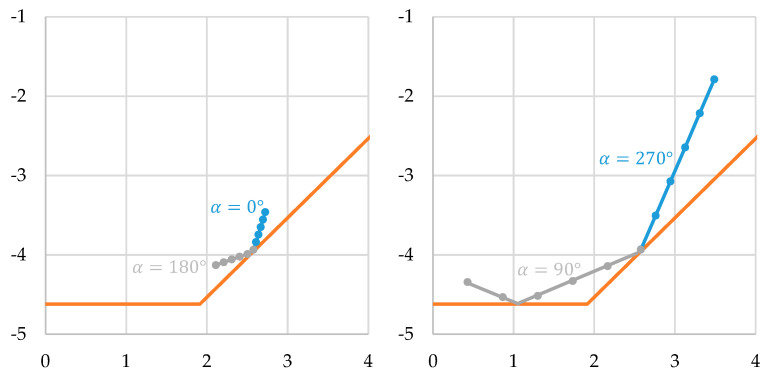
Position of the laser beam at the exit face in dependence of the bending angle (0≤β≤5°) and bending orientation α. Dots for each full degree of β. Dimensions are in mm.

The influence of the bending angle β for four exemplary orientations (angle α) is given in Figure 12. The bending intensity β varies between 0° and 5°, while each dot marks a full degree. The comparison of the left and right image in Figure 12 shows that the direction in which the laser beam moves does not clearly identify the bending direction α. In parallel, the displacement of the beam position gives no hint about the bending intensity β. This is not desirable because it makes it impossible to determine the bending parameters α and β and it is difficult to distinguish between torsion and bending. To overcome these limitations, more than one laser beam can be used. Several approaches are possible here, two of which will be discussed in the following section.

### 3.3. Combined Load Cases

**Figure 13:**
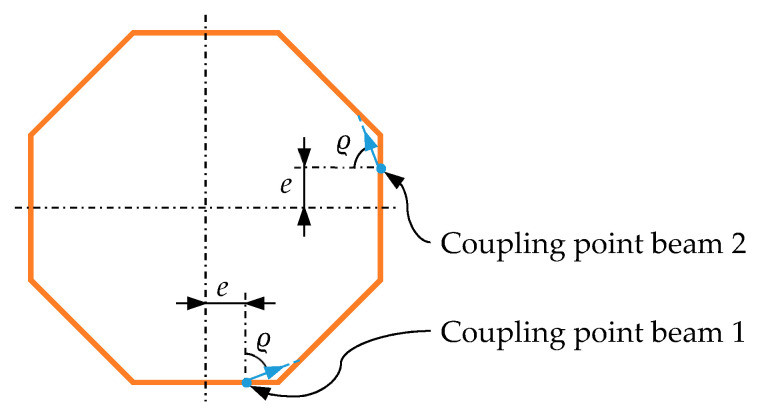
Coupling points of two laser beams at the first face of the prism rod.

**Figure 14:**
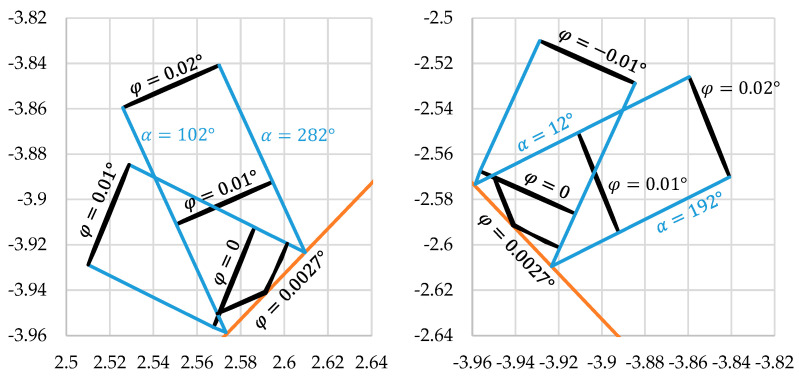
Bending ellipses (black) for different torsion angles φ with β=0.05°. **Left**: beam 1, **right**: beam 2. Dimensions are in mm.

With one laser, it is difficult or even impossible to separate between torsion and bending. One way to solve this problem is to double the existing beam path and to rotate it by 90° around the z-axis. While both lasers show the same coupling angle ε and offset e, the coupling position is altered (Figure 13). In this way, the sensitivity curve for bending (Figure 11) is also shifted by 90°. This increases the sensitivity for the bending directions that are difficult to measure, allows to determine the bending orientation and to distinguish between torsion and bending. The exit points of both laser beams are shown in Figure 14 for different values of α and φ with a constant bending angle (β=0.05°). The angles β and φ are chosen to be quite small, which represents a beginning deformation of the rod. The black “bending ellipses” result from varying α and keeping β constant (compare Figure 10). The blue curves in Figure 14 connect the far ends of the ellipses, where the sensitivity for bending is maximum. Each blue line corresponds to an angle α. Since the curves in Figure 8 represent the variation of φ with α=β=0, the blue curves here show the same orientation as in Figure 8 for small amplitudes of $\left|\varphi \right|$.

Comparing beams 1 and 2 (see Figure 13), the whole pattern in Figure 14 is rotated by 90°, as are the coupling points of the lasers. In this way, each combination of α, β and φ leads to a specific pattern of the two laser exit points so that a distinction between torsion and different bending directions is possible. Due to the complexity of the exit point movement under torsion (Figure 8) it is not clear if the patterns are always unique.

As discussed above, the bending sensitivity for the totally internal reflected laser beam is quite low. The reference beam used to determine the decency level, which propagates along the z-axis, can be used as a secondary beam as well. It offers increased and constant sensitivity for bending, independent of the angle α. The “bending ellipses” are now perfect circles. The bending intensity β can be easily derived from the distance of the laser from the middle point. Torsion does not influence the shape of the circles (does not affect the result of β), it only rotates them (superimposes with the bending direction α). Assuming a limited torsion angle ($\left|\varphi \right|$ is typically in the range of tenths of a degree or in its single-digit range), the angle α can be directly approximated. Based on these results, the interpretation of the totally internal reflected beam is much easier. With sufficient calibration data (see discussion in Section 4) the torsion angle φ can be determined. If necessary, the result for α can be corrected in an iterative procedure.

### 3.4. Sensitivity for Misalignment of the Laser

**Figure 15:**
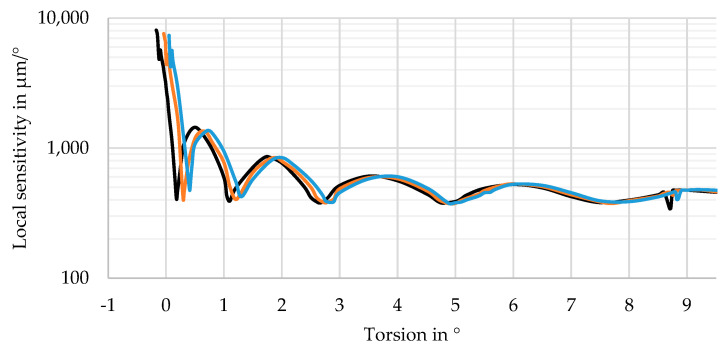
Influence of the exact orientation of the laser beam on the local sensitivity for torsion with e=1 mm. Lateral orientation of the laser beam input: black: ϱ=67.45°, orange: ϱ=67.5° (exact angle), blue: ϱ=67.55°.

For a cost-efficient sensor system, it is important that the components do not have to be adjusted too precisely without losing the functionality. Therefore, the lateral orientation of the laser at the input (angle ϱ in Figure 4 right) is varied by ±0.05° and the resulting local sensitivity for torsion analyzed (Figure 15). All other parameters are the same as in Section 3.1.3.

The sensitivity curve for the exact angle ϱ is given in orange in Figure 15. When changing this angle, the curves show an offset in φ-direction. The slight variations in the shape of the curves may be caused by the limited step size between the sample points used here. It varies according to the local curvature of the graphs between Δφ=0.02° and Δφ=0.5°. Changing the input direction from the exact value ϱ=67.5° by ±0.05° shifts the sensitivity curve by approximately ±0.1° in φ-direction. As a consequence, for ϱ=67.55° the laser is coupled out of the rod for φ<0.03°, so that a measurement at φ=0 is not possible. Accordingly, this effect can be used to fine-tune the systems range of high sensitivity.

### 3.5. Sensitivity for Temperature Changes

In addition to the deformation to be measured, temperature changes alter the shape of the rod. How this influence is reflected in the rod geometry strongly depends on the mechanical boundary conditions; i.e., on how the deformation is transferred from the measurement object to the rod. In this paper, unrestricted thermal extension in the longitudinal and transverse directions is assumed. The average thermal expansion coefficient of PMMA is $7.4\times 10^{-5}/K$ for a temperature range from 23 °C to 50 °C [39]. Furthermore, it is assumed that the detector does not change its size with varying temperature.

Two assemblies for the laser diode are possible, which change the influence of the temperature on the measurement results. The laser source can be attached to the rod, e.g., by gluing, and thus follow the movement of the expanding rod. In this case, the coupling position remains exactly at the edge of the rod, independent of the temperature. This concept is called “relative” here. The diode can also be connected to the measurement object, and thus not follow the movement of the rod. In this “absolute” case, the coupling position changes. With this concept, valid results are only possible for positive temperature changes ΔT. Otherwise, the laser beam will miss the shrunken rod.

**Figure 16:**
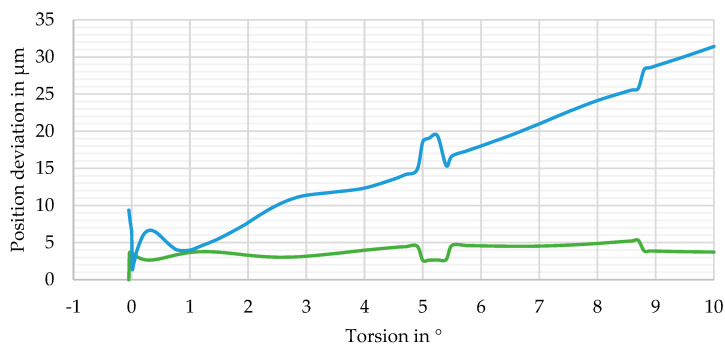
Influence of the thermal expansion (+10 K) of the rod on the exit position of the laser beam. Green: relative approach, blue: absolute approach.

To analyze the influence of temperature, the same parameters as in Section 3.1.3 are used. The resulting deviations of the laser exit point are illustrated in Figure 16 for the absolute and the relative assemblies. The temperature change is +10 K. For the relative setup, the exit position of the laser moves up to 5.3 µm, compared to its position at the reference temperature. This value is nearly independent from the actual torsion angle.

With the absolute assembly, the deviation almost linearly increases with increasing torsion angle. In both cases, the deviation curve suddenly changes at the points where the number of reflections of the laser inside the rod changes (compare Figure 8). In practice, the finite diameter of the laser will smoothen this effect. Since the relative approach lacks the linear component of the deviation, and thus, the influence of temperature on the measurement result is smaller, this arrangement is preferable. Therefore, to set up the sensor system, the laser source should be directly connected to the rod.

**Figure 17:**
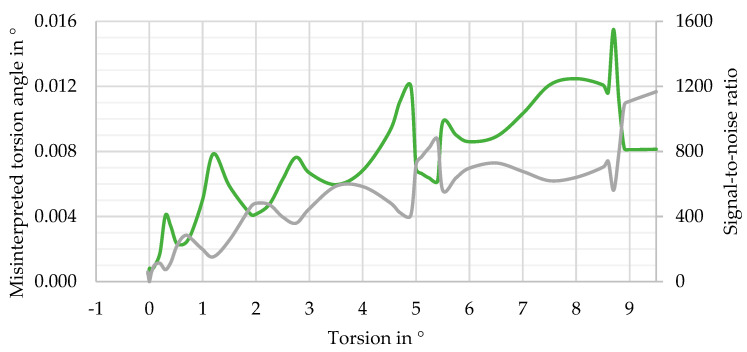
Possible misinterpretation of a temperature change of +10 K as torsion, relative approach. Green: angle in ° (left axis), grey: signal-to-noise ratio (right axis).

If the temperature change cannot be detected otherwise, it will be misinterpreted as deformation, e.g., as torsion. This “misinterpreted torsion angle” is calculated using the local sensitivity for torsion (Figure 9) and the position deviation from Figure 16. This approximation ignores the direction of the position change and, therefore, gives the most critical values. The resulting “misinterpreted torsion angles” are presented in Figure 17 (green curve) for the relative assembly and a temperature change of +10 K. The angles are quite small, below 0.0051°, for torsion angles in the range −0.03°<φ<1°.

The grey curve places the real torsion angle in relation to these results (signal-to-noise ratio). Therefore, it gives poor values in the vicinity of φ=0. For φ>0.4°, the temperature-induced deviation for +10 K is less than 1 % of the real torsion angle, leading to a signal-to noise ratio of more than 100. Depending on the measurement task, this small error can be ignored. For smaller torsion angles or greater temperature changes, the temperature should be monitored or its influence eliminated; e.g., by using multiple laser beams and an appropriate calibration.

## 4. Discussion

The simulation results attest that the sensor system has a high sensitivity for torsion if the laser beam is not coupled into the rod at the middle of a lateral face. Using more than one laser, different load cases can be distinguished.

Two aspects make the interpretation of the results on the detector a challenge. On the one hand, torsion leads to complex movements of the totally internal reflected laser beam on the detector (see Section 3.3). The beam path is additionally influenced by other deformations, such as bending and temperature changes (Section 3.5). On the other hand, small deviations of the input parameters, such as the orientation of the laser beam, influence the exit point and its movement as well (Section 3.4). This leads to the following conclusions: First, it is hard to simulate the exit point of the laser for all possible load cases, temperature conditions and manufacturing tolerances in advance. Therefore, the whole exit face should be considered as a potential outcoupling location and covered with the 2D detector. Second, the sensor system has to be calibrated. The use of AI for data evaluation can be advantageous here as it is currently investigated for the identification of dynamic loads on structural components [40,41].

So far, only a limited number of rod geometries have been analyzed. As shown in this article, TIR prism rods can be used as highly sensitive measurement systems and optimized for certain load cases, as shown here for torsion. Nevertheless, other cross-section shapes have to be investigated and different coupling points need to be analyzed in order to find an optimum between the sensitivity for the desired load case, the influence of other load types on the results and the linearity of the measurement signal. Furthermore, manufacturing and further positioning tolerances have to be included in the optimization process, as well as a finite diameter of the laser beam and possible thermal expansion. The finite spot size of the laser will lead to extended spot geometries on the detector, since the lateral faces of the rod show a specific curvature under bending and torsion.

Bonenberger et al. suggested using prism rods with convex-shaped lateral faces to improve the rods light mixing properties [42] (the definition “convex” refers to the light propagating inside the rod; the faces appear concave as the rod is seen from the outside). In this way, rays that are reflected multiple times by the lateral faces propagate almost chaotical. Small deviations in the input parameters, such as the position or the angle of the rays, lead to significant changes at the output. The same approach can be used here to further increase the sensitivity of the system or to reduce the required number of reflections to realize a specific sensitivity. This reduces the system size and the sensitivity for tolerances. However, it has to be considered that using curved lateral faces for the reflection will automatically increase the laser spot size at the detector, which then forms a line. The smaller the radius of the curvature of the lateral faces, the higher the sensitivity of the system. In the same way, the line length on the detector is increased. This effect can be reduced using one of the following approaches. By combining convex- and concave-shaped lateral faces, the laser spot size can be limited. Alternatively, a basic design principle of multi-pass cells can be used here as well. In Herriott-type cells [26], for example, the laser beam is reflected back and forth several times between two concave-shaped mirrors and is focused on the virtual plane in their center. By adjusting the shape of the lateral faces, focus points of the laser can be generated inside the rod in a similar way, keeping the spot size at the detector small.

As a future research topic, the influence of birefringence on the measurement results has to be investigated in more detail. For example, a material without orientation birefringence, but with photoelastic birefringence, can increase the sensitivity of the sensor to small deformations. When using such a material for the rod, no birefringence occurs in the undeformed case. With increasing deformation, the birefringence becomes more obvious and leads to an enlarged laser spot on the detector. In this way, the position and the size change can be used as measurement data.

## Figures and Tables

**Table 1:** Number of reflections $n_{r}$ local sensitivity σ and gain factor $\sigma /\hat{\sigma }$ at φ=0 for different numbers of faces n and coupling angles ε.

n	ε in °	$n_{r}$	σ in µm/°	$\sigma /\hat{\sigma }\;$
6	60	48	63 *	0.72 *
	70	76	153	1.75
	80	157	76	0.87
8	60	58	83	0.95
	70	93	85	0.97
	80	192	90	1.03

* These values are hard to exactly determine and, therefore, show an uncertainty, since the exit point of the laser is close to an edge of the rod in this case (compare Figure 8).

**Table 2:** Number of reflections $n_{r}$ local sensitivity σ and gain factor $\sigma /\hat{\sigma }$ at φ=0 for different numbers of faces n and coupling angles ε decentral coupling (e=1 mm).

n	ε in °	$n_{r}$	σ in µm/°	$\sigma /\hat{\sigma }\;$
6	60	48	1403 *	16.08 *
	70	76	2267	25.98
	80	157	4733	54.24
8	60	58	1863	21.35
	70	93	2971	34.05
	80	192	4804 *	55.05 *

* These values are hard to exactly determine and, therefore, show an uncertainty, since the exit point of the laser is close to an edge of the rod in these cases (compare Figure 8).

## Data Availability

All data generated or analyzed that are relevant for the findings of this paper are presented here. Due to the simple geometry of the prism rod, it can easily be reproduced with appropriate software tools.
